# Perspectives on Understanding Aberrant Brain Networks in Epilepsy

**DOI:** 10.3389/fnetp.2022.868092

**Published:** 2022-04-05

**Authors:** Nishant Sinha, Rasesh B. Joshi, Mani Ratnesh S. Sandhu, Theoden I. Netoff, Hitten P. Zaveri, Klaus Lehnertz

**Affiliations:** ^1^ Department of Neurology, Penn Epilepsy Center, Perelman School of Medicine, University of Pennsylvania, Philadelphia, PA, United States; ^2^ Center for Neuroengineering and Therapeutics, University of Pennsylvania, Philadelphia, PA, United States; ^3^ Boston Children’s Hospital and Harvard Medical School, Boston, MA, United States; ^4^ Department of Laboratory Medicine, Yale University, New Haven, CT, United States; ^5^ Department of Biomedical Engineering, University of Minnesota, Minneapolis, MN, United States; ^6^ Department of Neurology, Yale University, New Haven, CT, United States; ^7^ Department of Epileptology, University of Bonn Medical Centre, Bonn, Germany; ^8^ Helmholtz Institute for Radiation and Nuclear Physics, University of Bonn, Bonn, Germany; ^9^ Interdisciplinary Center for Complex Systems, University of Bonn, Bonn, Germany

**Keywords:** brain network, epilepsy, treatment, network aberrance, network models

## Abstract

Epilepsy is a neurological disorder affecting approximately 70 million people worldwide. It is characterized by seizures that are complex aberrant dynamical events typically treated with drugs and surgery. Unfortunately, not all patients become seizure-free, and there is an opportunity for novel approaches to treat epilepsy using a network view of the brain. The traditional seizure focus theory presumed that seizures originated within a discrete cortical area with subsequent recruitment of adjacent cortices with seizure progression. However, a more recent view challenges this concept, suggesting that epilepsy is a network disease, and both focal and generalized seizures arise from aberrant activity in a distributed network. Changes in the anatomical configuration or widespread neural activities spanning lobes and hemispheres could make the brain more susceptible to seizures. In this perspective paper, we summarize the current state of knowledge, address several important challenges that could further improve our understanding of the human brain in epilepsy, and invite novel studies addressing these challenges.

## Spatial and Temporal Aspects of Brain Networks

Network neuroscience has transformed our conceptualization of the brain as a complex network (Bullmore and Sporns, 2009; Bassett and Sporns, 2017). A complex network consists of vertices and edges and has non-trivial topological features (Boccaletti et al., 2006; Cohen and Havlin, 2010). Depending on the chosen spatial scale, a vertex may represent a cell or a brain region, and an edge represents some connection between vertices. Brain networks are mainly of two types: structural and functional. In a structural network, an edge represents a physical/anatomical connection between vertices. In a functional network, an edge represents some functional interaction between vertices. By conceptualizing the brain as a network, we can study its topological and other network-related properties quantitatively by employing a wide range of methods from graph theory (Fornito et al., 2016; Newman, 2018; Chung, 2019).

Brain networks can be defined at and across multiple levels of spatial and temporal scales. There are three broadly recognized spatial scales: microscale, mesoscale, and macroscale. At these spatial scales, the definition of vertices changes to either single cells (e.g., neurons), group of cells (e.g., cortical columns), or brain regions (e.g., parcellated areas). For structural brain networks, edges at microscale, mesoscale, and macroscale could be defined as single synapses, fiber bundles, or groups of fiber bundles. The edges are almost always defined for functional brain networks based on properties of some interaction between activities recorded at the vertices. These properties can be characterized with a wide range of time-series-analysis techniques (Lehnertz et al., 2020) and define the type of network at different scales, such as directed/undirected and binary/weighted networks. The timespan determines the definition of networks ranging from seconds to days or changes over lifespan via plasticity mechanisms. In such evolving (time-dependent/temporal/multiplex) networks (Bullmore and Sporns, 2009) not only the number of vertices and edges may change over time, but also the properties of edges can evolve. The spatial-temporal network architecture of the human brain can illuminate how human cognition and behavior emerges (Medaglia et al., 2015), how they vary over development and aging (Damoiseaux, 2017), and its alteration in disease or injury (Liu et al., 2017).

The best practices for constructing and analyzing brain networks are still evolving with many unknowns and challenges. Limiting our focus on epilepsy, in this perspective, our objective is to summarize the current state of knowledge and highlight the areas that need to be addressed in future studies to further improve the understanding of aberrant brain networks in epilepsy.

## Network Aberrance

Conceptualizing the human brain as a network has led to a paradigm shift in establishing epilepsy as a network disorder (Spencer, 2002; Berg and Scheffer, 2011; Kramer and Cash, 2012; Engel et al., 2013; Lehnertz et al., 2014; Bernhardt et al., 2015; Stacey et al., 2020). A network representation of epilepsy offers a powerful framework to understand how seizures originate, propagate, and terminate. The complex network structure of the brain emerges during development through a process of creating connections and refining those connections. The resulting structural network allows for rich dynamics and information processing. Seizures may emerge due to subtle changes in this network. Altered connections between areas can result in a change in stability that enables the emergence and propagation of seizures. These changes may occur with or without changes in excitability in the network. Seizures, even in patients with epilepsy, only represent a small fraction of the dynamic activity observed in the brain. An epileptic network is not necessarily seizing, it is a network in which the propensity to seize is higher than normal. It is possible that all brain networks, under the right conditions, may seize. Therefore, an aberrant network can be defined by an increased probability of a seizure.

### Vertex and Edge Aberrance

There are four possible causes for network aberrance (Gummadavelli et al., 2018): vertex, edge, vertex-edge, and emergent aberrance. a) Vertex aberrance: Most prior work in epilepsy conceived aberrance to arise from a seizure focus, which can be regarded as a vertex (or a group of vertices). These prior studies reported abnormalities in cell dynamics caused by channelopathies (van Loo and Becker, 2020), or changes in the structure of a brain region, such as a focal cortical dysplasia (Gill et al., 2021; Sinha and Davis, 2021) or hippocampal sclerosis (Winston et al., 2013; Chen et al., 2018). b) Edge aberrance: Altered connections between neurons or areas can be caused by changes in conduction velocities, which could occur due to changes in myelination, or by changes in synaptic dynamics. Edge-centric approaches have been developed to capture these abnormalities, e.g., traumatic axonal injuries in traumatic brain injury (TBI) or abnormally altered functional connections between regions inside and outside the epileptogenic tissues (Diaz-Arrastia et al., 2014; Rings et al., 2019; Yue et al., 2019; Fruengel et al., 2020; Graham et al., 2020). c) Vertex-edge aberrance: It is plausible that abnormalities in vertices and connected edges influence each other, i.e., network aberrance can result from a conjunction of both edge and vertex aberrance (Rings et al., 2019; Fruengel et al., 2020). d) Emergent aberrance: In emergent aberrance, even though the individual vertices and edges of the network are not aberrant, aberrance arises because of the network’s dynamics and/or topology. An open question is whether a normal non-epileptic network, recruited into epileptiform activity is part of emergent network aberrance (Sloviter, 2008).

### Epileptogenesis vs. Ictogenesis

Network aberrance is associated with the pathophysiological mechanism underlying epileptogenesis and ictogenesis. Epileptogenesis refers to the mechanism by which epilepsy develops i.e., when healthy brain networks develop the propensity to generate seizures (Goldberg and Coulter, 2013; Pitkänen et al., 2015; Bartolomei et al., 2017; Sinha et al., 2019; Baruah et al., 2020). For example, increased risk of epileptogenesis after traumatic brain injury might be associated with aberrance in brain networks due to injury in major white-matter fasculli (Pitkänen et al., 2016). Ictogenesis refers to the development of seizures in patients with epilepsy i.e., the mechanism by which seizures originate in brain networks (Blauwblomme et al., 2014; Wolf and Beniczky, 2014; Paz and Huguenard, 2015). Recent studies show that even in the same epilepsy patient, the functional brain network may evolve seizures specifically (Schroeder et al., 2020). Therefore, network aberrance associated with epileptogenesis and ictogenesis could be highly patient-specific and might evolve with disease progression in individual patients.

### Heterogeneities

Seizures can arise out of a myriad of pathologic substrates, resulting in significant heterogeneity among patient populations. This poses significant challenges in studying the mechanisms that give rise to an epileptic network and in developing individualized therapeutic interventions. Heterogeneities between patients can include age (Guerrini, 2006; Thijs et al., 2019; Sen et al., 2020), sex, genetic differences, structural and anatomic differences, comorbidities, medications (Löscher et al., 2013; Haneef and Chiang, 2014), amongst others. For example, in psychiatric comorbidities, it is well-known that the prevalence of conditions like depression and anxiety are significantly higher in patients with epilepsy than the general population, however, the reason for this relationship is unclear. A widely used approach to studying epilepsy is based on a case-control design, i.e., isolating a variable of interest, grouping patients based on this variable, and comparing to a control population (Marquand et al., 2016a; Marquand et al., 2016b; Marquand et al., 2019). This case-control design can be expanded to understand how individual network characteristics can be defined with multimodal data (structural and functional imaging, electrophysiology, genomics, etc.) for mapping individual patients on disease spectrum to tailor patient-specific therapies.

## Existing Techniques to Control or Correct Network Aberrance

A network aberrance may be treated by lesioning connections, stimulating brain areas in the network to promote plasticity and reweighting of the connections, or to change the homeostatic setpoints maintaining the connections within the network. Treating an abnormal epileptic network requires a patient-specific understanding of the brain network to develop an optimal treatment plan.

### Surgery

Epilepsy surgery is an effective therapy to control drug-resistant seizures, with nearly 50% patients achieving seizure freedom after surgery. Epilepsy surgery introduces a specific change to normalize the aberrant epileptic network (Sinha et al., 2014; Sinha et al., 2016; Sinha et al., 2017; Taylor et al., 2018; Ramaraju et al., 2020; Wang et al., 2020; Sinha et al., 2021a; Bernabei et al., 2022; Taylor et al., 2022). There is a critical need to develop methods to quantitatively map brain network aberrance before surgery and validate these quantitative measures with surgical decisions and outcomes.

### Stimulation

Devices, more recently, have emerged as the third line of treatment after pharmacotherapy and resective or disconnective surgery. The implanted device may stimulate in an open-loop manner or monitor brain signals from electrode contacts and send electrical pulses through the electrode contacts to the brain in a closed-loop response to aberrant electrical signals to disrupt the emerging seizure. The use of invasive electrical stimulation devices is an active topic of investigation, and research is underway to determine optimal stimulation parameters. First-generation implanted devices were not designed to monitor and modulate brain networks (Gummadavelli et al., 2018). Instead, they were designed for monitoring and controlling activity at one or a few brain locations. To be able to monitor and modulate a brain network would entail being able to monitor and stimulate multiple brain areas with real-time evaluation of relationship measures and understanding when, where, and how to stimulate to achieve network modulation. This may require identifying “hubs” or “choke points” or strategic regions in the network that can terminate abnormal activity or prevent activity from propagating through the network. In addition, we may need to develop a novel stimulation paradigm adapted to the extended spatiotemporal network nature of the brain, develop a better map of the dynamic states of the network, and understand the stimuli needed to tailor the neuromodulation therapy for an individual. As an alternative to implanted devices, devices can be used to non-invasively stimulate networks, such as transcranial magnetic stimulation (TMS) (Badawy et al., 2014; Tsuboyama et al., 2020; Vlachos et al., 2022), transcranial direct current stimulation (tDCS) (Tecchio et al., 2018), or transcutaneous auricular vagus nerve stimulation (taVNS) (von Wrede et al., 2021; Rings et al., 2021). These approaches can be used for global network activation rather than stimulating specific vertices or edges within the network.

### Behavior and Cognition

In addition to the above established control techniques, cognitive and behavioral treatments for epilepsy offer several advantages: they are relatively low cost and noninvasive, lack serious side effects, and facilitate patient participation (Tang et al., 2014; Leeman-Markowski and Schachter, 2017; Nagai, 2019; Thijs, 2019). One of these approaches – biofeedback – employs behavioral control strategies based on operant conditioning to regulate physiological activity. Through visual and auditory feedback, patients learn how to voluntarily modulate – in real time – physiological responses, such as heart rate, respiration, electrodermal or electroencephalographic activity. Biofeedback is widely assumed to act by influencing thalamocortical regulation. Electrodermal activity (EDA) biofeedback has been shown to significantly reduce seizure frequency, and the post-therapy seizure reduction correlated linearly with enhanced interactions between frontal brain regions known to mediate attentional and executive functions in feedback learning and cognitive control (Nagai et al., 2018). Another study provided evidence of an involvement of the occipital cortices that process visual information as well as of cortical and subcortical areas that are associated with interoceptive awareness (Critchley, 2002). Overall, EDA biofeedback appears to elicit distributed but diffuse and unspecific network activity (Schach et al., 2022). A better understanding of the mechanism of action of cognitive and behavioral approaches may help establish these techniques as additional or alternative non-pharmaceutical treatment options and may help to improve understanding of neurobehavioural comorbidities of epilepsy (Hermann et al., 2021).

### Drugs and Diet

Although anti-seizure medications and their long-term effects on the brain is a broad field of literature unto itself, the development of specific measures to quantify effects of individual medications in a patient-specific manner is of particular interest. Broadly, the effects of anti-seizure medications are generally conceptualized as tipping the balance between excitation and inhibition, and we are beginning to reconcile this understanding with the modulation of aberrant synchrony and network architecture in epilepsy. To this end, several studies have started to address this question by developing measures to quantify medication-related changes (Meisel, 2020)*.* Many open questions remain, including quantifying long-term effects of medications on network architecture and assessing and/or predicting treatment response based on network configurations.

Many drug-resistant epilepsy patients are not candidates for surgery, leading to consideration for alternate and non-pharmacologic treatments, such as the ketogenic diet (KD). The classic ketogenic diet consists of a high-fat, and low carbohydrate diet, switching the body’s metabolism to consume ketones as a primary fuel source (Rho, 2017; D’Andrea Meira et al., 2019). The mechanisms behind the efficacy of KD are still being explored with emerging evidence suggesting that the anti-seizure properties of KD may be mediated by an enrichment of specific KD-associated gut microbiota (Olson et al., 2018). It is possible that KD may also exert its effects by modulating epileptic networks independently or via the gut microbiota. A recent study showed that changing predominant dietary fuel from glucose to ketones increases sustained functional communication between brain regions, i.e., increases brain network stability (Mujica-Parodi et al., 2020). Another study showed that modulating the gut microbiome can change brain-wide functional connectivity and structural organization (Aswendt et al., 2021). Therefore, KD can influence brain networks directly via ketosis or by modulating gut microbiota representing a growing need to study the effects of KD on networks in the context of epilepsy.

## What Are the Future Tools Existing and Conceptual Tools?

### Measurement Tools

The development of the field of network neuroscience challenges us to develop new measurement tools and methods. To better understand the development of network aberrance, we need to be able to monitor the vertices and edges of brain networks, at different spatial scales, with structural and functional modalities and over time. We need to make measurements over long durations to allow for the capture and determination of relationships between different network vertices. We also need to capture information with different modalities which can let us infer the electrophysiological, neurochemical, and metabolic network alterations which accompany epileptogenesis and ictogenesis. While progress has been made with functional modalities such as fMRI BOLD and electrophysiology, and there have been clear demonstrations of network changes in epilepsy with these modalities, challenges exist in combining information on networks determined from these modalities. Challenges also exist, in combining information from functional and structural imaging modalities. These different challenges call for the development of new sensor technologies for continuous measurement, new multimodal brain probes, new methods for imaging the brain, and new conceptual and mathematical approaches for fusing multimodal data and studying network activity (Spencer et al., 2018).

### Normative Approach

Many recent studies are adopting the normative modeling approach, which is a case-control method that leverages huge control databases for quantifying deviations in individual patients (Frauscher et al., 2018; Bernabei et al., 2022; Taylor et al., 2022). In this approach, measurement from a patient’s brain networks is standardized against equivalent measurement derived from a group of controls. Thus, the aberrance in the patient’s network is quantified as a deviation from the normal range expected in controls. Normative modeling approach is routinely applied across a range neurological and psychiatric disorders (Marquand et al., 2016a; Marquand et al., 2016b; Marquand et al., 2019; Sinha et al., 2021a; Sinha et al., 2021b). In epilepsy, recent studies applied the normative modeling approach for mapping abnormalities remaining after epilepsy surgery using structural brain networks and mapping epileptogenic tissues using normative intracranial EEG atlases using functional brain networks (Sinha et al., 2021a; Sinha et al., 2021b; Bernabei et al., 2022; Taylor et al., 2022). These approaches can potentially reconcile heterogeneities and stratify patients on a disease spectrum enabling patient-specific therapies.

### Computational Models of Networks

High density recordings from cortex and depth electrodes have provided amazing opportunities to study the dynamics of brain areas and functional connections between them. But the high dimensionality of the data makes it difficult to interpret the origins of aberrant activity. Computational models of neuronal networks enable probing the parameters and states to better understand the dynamics and how vertex dynamics and network structure affects brain dynamics. Models can be fit to recorded neuronal data to infer changes in the connection structures, dynamics, and hidden states of the brain. Hodgkin-Huxley-type models are used to simulate neurons (Hodgkin and Huxley, 1990) and mean-field models, such as the Jansen-Rit model can be used to simulate local field potentials (Jansen and Rit, 1995). Mean-field models can easily be extended to incorporate slow dynamics to simulate ictogenesis and seizure termination (Jirsa et al., 2014) or scaled up to make whole brain scale networks using anatomical connections (Sanz Leon et al., 2013; Falcon et al., 2016).

The development of modeling platforms, such as Neuron (Nicholas and Michael, 2010), and platforms specifically to model networks, such as PyNN (Davison et al., 2008) and The Virtual Brain (Falcon et al., 2016), have facilitated the ease and entry into using simulations to understand brain dynamics. Repositories of models, such as ModelDB (McDougal et al., 2017), allow sharing of existing models. These predictive models can be used to design closed-loop control to modulate states and functional connections within the brain.

### Dynamical Systems Analysis Tools

Understanding dynamics of brain networks requires an understanding of the dynamics of vertices and the network structure together. Determining what makes a network stable or unstable requires dynamical systems analysis tools. There are well developed tools for understanding the dynamics of linear and time-invariant (LTI) systems in MATLAB and Python. However, neuronal dynamics are nonlinear and non-stationary. But, with most nonlinear systems, they often act linearly locally in state space to small perturbations and LTI systems analysis can be very informative and should never be underestimated. There are excellent nonlinear analysis tools that can be extremely valuable in characterizing complex dynamical systems, such as XPP/XPPAut (Ermentrout and Mahajan, 2003), which can be used to identify bifurcations as a function of parameters (e.g., input drive or interaction strength between areas), of brain models. Sophisticated network analysis tools have also been developed, such as the Brain Connectivity Toolbox (Rubinov and Sporns, 2010).

## Conclusion

Network studies of the human brain and epilepsy remain at an early stage and much about the aberrance of brain networks in epilepsy remains poorly understood. In this article we have delineated some of the open areas of research. We invite studies in these areas and other topics on brain network disorders ranging from the best practices in characterizing epileptic networks and its constituents, modeling to illustrate the pathophysiological mechanisms, techniques to control network aberrance and models integrating different spatial and temporal resolutions to illustrate emergent network aberrance associated with epileptogenesis and ictogenesis. We encourage longitudinal studies combining multimodal imaging, electrophysiology, and genetics to identify biomarkers for diagnosis and prognosis of medication resistance, treatment outcomes, effects of ketogenic diet on networks in the context of epilepsy, broader questions on comorbidities, neurological and psychiatric brain network disorders, interactions between brain networks and the gut microbiome and other metabolic processes.

## Data Availability

The original contributions presented in the study are included in the article/Supplementary Material, further inquiries can be directed to the corresponding author.

## References

[B1] Aswendt M. Green C. Sadler R. Llovera G. Dzikowski L. Heindl S. (2021). The Gut Microbiota Modulates Brain Network Connectivity under Physiological Conditions and after Acute Brain Ischemia. Iscience 24 (10), 103095. 10.1016/j.isci.2021.103095 34622150 PMC8479691

[B2] Badawy R. A. B. Strigaro G. Cantello R. (2014). TMS, Cortical Excitability and Epilepsy: The Clinical Impact. Epilepsy Res. 108 (2), 153–161. 10.1016/j.eplepsyres.2013.11.014 24315665

[B3] Bartolomei F. Lagarde S. Wendling F. McGonigal A. Jirsa V. Guye M. (2017). Defining Epileptogenic Networks: Contribution of SEEG and Signal Analysis. Epilepsia 58 (7), 1131–1147. 10.1111/epi.13791 28543030

[B4] Baruah J. Vasudevan A. Köhling R. (2020). Vascular Integrity and Signaling Determining Brain Development, Network Excitability, and Epileptogenesis. Front. Physiol. 10, 1583. 10.3389/fphys.2019.01583 32038280 PMC6987412

[B5] Bassett D. S. Sporns O. (2017). Network Neuroscience. Nat. Neurosci. 20 (3), 353–364. 10.1038/nn.4502 28230844 PMC5485642

[B6] Berg A. T. Scheffer I. E. (2011). New Concepts in Classification of the Epilepsies: Entering the 21st century. Epilepsia 52 (6), 1058–1062. 10.1111/j.1528-1167.2011.03101.x 21635233

[B7] Bernabei J. M. Sinha N. Arnold T. C. Conrad E. (2022). Normative Intracranial EEG Maps Epileptogenic Tissues in Focal Epilepsy. Brain. 10.1093/brain/awab480 PMC9630716 35640886

[B8] Bernhardt B. C. Bonilha L. Gross D. W. (2015). Network Analysis for a Network Disorder: The Emerging Role of Graph Theory in the Study of Epilepsy. Epilepsy Behav. 50, 162–170. 10.1016/j.yebeh.2015.06.005 26159729

[B9] Blauwblomme T. Jiruska P. Huberfeld G. (2014). Mechanisms of Ictogenesis. Int. Rev. Neurobiol. 114, 155–185. 10.1016/b978-0-12-418693-4.00007-8 25078502

[B10] Boccaletti S. Latora V. Moreno Y. Chavez M. Hwang D. (2006). Complex Networks: Structure and Dynamics. Phys. Rep. 424 (4-5), 175–308. 10.1016/j.physrep.2005.10.009

[B11] Bullmore E. Sporns O. (2009). Complex Brain Networks: Graph Theoretical Analysis of Structural and Functional Systems. Nat. Rev. Neurosci. 10 (3), 186–198. 10.1038/nrn2575 19190637

[B12] Chen C. Li H. Ding F. Yang L. Huang P. Wang S. (2018). Alterations in the Hippocampal-Thalamic Pathway Underlying Secondarily Generalized Tonic-Clonic Seizures in Mesial Temporal Lobe Epilepsy: A Diffusion Tensor Imaging Study. Epilepsia 60 (1), 121–130. 10.1111/epi.14614 30478929

[B13] Chung Moo. K. (2019). Brain Network Analysis. Cambridge University Press.

[B14] Cohen Reuven. Havlin Shlomo. (2010). Complex Networks: Structure, Robustness and Function. Cambridge University Press.

[B15] Critchley H. D. (2002). Review: Electrodermal Responses: What Happens in the Brain. Neuroscientist 8 (2), 132–142. 10.1177/107385840200800209 11954558

[B16] Damoiseaux J. S. (2017). Effects of Aging on Functional and Structural Brain Connectivity. Neuroimage 160, 32–40. 10.1016/j.neuroimage.2017.01.077 28159687

[B17] D’Andrea Meira I. Romão T. T. Pires do Prado H. J. Krüger L. T. Pires M. E. P. da Conceição P. O. (2019). Ketogenic Diet and Epilepsy: What We Know So Far. Front. Neurosci. 13, 5. 10.3389/fnins.2019.00005 30760973 PMC6361831

[B18] Davison A. P. Brüderle D. Eppler J. (2008). PyNN: a Common Interface for Neuronal Network Simulators. Front. Neuroinform. 2, 11. 10.3389/neuro.11.011.2008 19194529 PMC2634533

[B19] Diaz-Arrastia R. Wang K. K. W. Papa L. Sorani M. D. Yue J. K. Puccio A. M. (2014). Acute Biomarkers of Traumatic Brain Injury: Relationship between Plasma Levels of Ubiquitin C-Terminal Hydrolase-L1 and Glial Fibrillary Acidic Protein. J. Neurotrauma 31 (1), 19–25. 10.1089/neu.2013.3040 23865516 PMC3880090

[B20] Engel J. Jr Jr. P. M. Thompson P. M. Stern J. M. Staba R. J. Bragin A. (2013). Connectomics and Epilepsy. Curr. Opin. Neurol. 26 (2), 186–194. 10.1097/wco.0b013e32835ee5b8 23406911 PMC4064674

[B21] Ermentrout B. Mahajan A. (2003). Simulating, Analyzing, and Animating Dynamical Systems: A Guide to XPPAUT for Researchers and Students. Appl. Mech. Rev. 56 (4), B53. 10.1115/1.1579454

[B22] Falcon M. I. Jirsa V. Solodkin A. (2016). A New Neuroinformatics Approach to Personalized Medicine in Neurology: The Virtual Brain. Curr. Opin. Neurol. 29 (4), 429–436. 10.1097/wco.0000000000000344 27224088 PMC5536184

[B23] Fornito A. Zalesky A. Bullmore E. T. (2016). Fundamentals of Brain Network Analysis. Amsterdam: Academic Press. 10.1016/b978-0-12-407908-3.00003-0

[B24] Frauscher B. von Ellenrieder N. Zelmann R. Doležalová I. Minotti L. Olivier A. (2018). Atlas of the normal Intracranial Electroencephalogram: Neurophysiological Awake Activity in Different Cortical Areas. Brain 141 (4), 1130–1144. 10.1093/brain/awy035 29506200

[B25] Fruengel R. Bröhl T. Rings T. Lehnertz K. (2020). Reconfiguration of Human Evolving Large-Scale Epileptic Brain Networks Prior to Seizures: an Evaluation with Node Centralities. Sci. Rep. 10 (1), 21921. 10.1038/s41598-020-78899-7 33318564 PMC7736584

[B26] Gill R. S. Lee H. M. Caldairou B. Hong S. J. Barba C. Deleo F. (2021). Multicenter Validation of a Deep Learning Detection Algorithm for Focal Cortical Dysplasia. Neurology 97 (16), e1571–e1582. 10.1212/WNL.0000000000012698 34521691 PMC8548962

[B27] Goldberg E. M. Coulter D. A. (2013). Mechanisms of Epileptogenesis: a Convergence on Neural Circuit Dysfunction. Nat. Rev. Neurosci. 14 (5), 337–349. 10.1038/nrn3482 23595016 PMC3982383

[B28] Graham N. S. N. Jolly A. Zimmerman K. Bourke N. J. Scott G. Cole J. H. (2020). Diffuse Axonal Injury Predicts Neurodegeneration after Moderate-Severe Traumatic Brain Injury. Brain 143 (12), 3685–3698. 10.1093/brain/awaa316 33099608

[B29] Guerrini R. (2006). Epilepsy in Children. The Lancet 367 (9509), 499–524. 10.1016/s0140-6736(06)68182-8 16473127

[B30] Gummadavelli A. Zaveri H. P. Spencer D. D. Gerrard J. L. (2018). Expanding Brain-Computer Interfaces for Controlling Epilepsy Networks: Novel Thalamic Responsive Neurostimulation in Refractory Epilepsy. Front. Neurosci. 12, 474. 10.3389/fnins.2018.00474 30108472 PMC6079216

[B31] Haneef Z. Chiang S. (2014). Clinical Correlates of Graph Theory Findings in Temporal Lobe Epilepsy. Seizure 23 (10), 809–818. 10.1016/j.seizure.2014.07.004 25127370 PMC4281255

[B32] Hermann B. P. Struck A. F. Busch R. M. Reyes A. Kaestner E. McDonald C. R. (2021). Neurobehavioural Comorbidities of Epilepsy: towards a Network-Based Precision Taxonomy. Nat. Rev. Neurol. 17 (12), 731–746. 10.1038/s41582-021-00555-z 34552218 PMC8900353

[B33] Hodgkin A. L. Huxley A. F. (1990). A Quantitative Description of Membrane Current and its Application to Conduction and Excitation in Nerve. Bltn Mathcal Biol. 52 (1-2), 25–71. 10.1007/bf02459568 2185861

[B34] Jansen B. H. Rit V. G. (1995). Electroencephalogram and Visual Evoked Potential Generation in a Mathematical Model of Coupled Cortical Columns. Biol. Cybern. 73 (4), 357–366. 10.1007/bf00199471 7578475

[B35] Jirsa V. K. Stacey W. C. Quilichini P. P. Ivanov A. I. Bernard C. (2014). On the Nature of Seizure Dynamics. Brain 137 (8), 2210–2230. 10.1093/brain/awu133 24919973 PMC4107736

[B36] Kramer M. A. Cash S. S. (2012). Epilepsy as a Disorder of Cortical Network Organization. Neuroscientist 18 (4), 360–372. 10.1177/1073858411422754 22235060 PMC3736575

[B37] Leeman-Markowski B. A. Schachter S. C. (2017). Cognitive and Behavioral Interventions in Epilepsy. Curr. Neurol. Neurosci. Rep. 17 (5), 42. 10.1007/s11910-017-0752-z 28382495 PMC6094950

[B38] Lehnertz K. Ansmann G. Bialonski S. Dickten H. Geier C. Porz S. (2014). Evolving Networks in the Human Epileptic Brain. Physica D: Nonlinear Phenomena 267, 7–15. 10.1016/j.physd.2013.06.009

[B39] Lehnertz K. Bröhl T. Rings T. (2020). The Human Organism as an Integrated Interaction Network: Recent Conceptual and Methodological Challenges. Front. Physiol. 11, 598694. 10.3389/fphys.2020.598694 33408639 PMC7779628

[B40] Liu J. Li M. Pan Y. Lan W. Zheng R. Wu F.-X. (2017). Complex Brain Network Analysis and its Applications to Brain Disorders: A Survey. Complexity 2017, 1–27. 10.1155/2017/8362741

[B41] Löscher W. Klitgaard H. Twyman R. E. Schmidt D. (2013). New Avenues for Anti-epileptic Drug Discovery and Development. Nat. Rev. Drug Discov. 12 (10), 757–776. 10.1038/nrd4126 24052047

[B42] Marquand A. F. Kia S. M. Zabihi M. Wolfers T. Buitelaar J. K. Beckmann C. F. (2019). Conceptualizing Mental Disorders as Deviations from Normative Functioning. Mol. Psychiatry 24 (10), 1415–1424. 10.1038/s41380-019-0441-1 31201374 PMC6756106

[B43] Marquand A. F. Rezek I. Buitelaar J. Beckmann C. F. (2016). Understanding Heterogeneity in Clinical Cohorts Using Normative Models: Beyond Case-Control Studies. Biol. Psychiatry 80 (7), 552–561. 10.1016/j.biopsych.2015.12.023 26927419 PMC5023321

[B44] Marquand A. F. Wolfers T. Mennes M. Buitelaar J. Beckmann C. F. (2016). Beyond Lumping and Splitting: A Review of Computational Approaches for Stratifying Psychiatric Disorders. Biol. Psychiatry Cogn. Neurosci. Neuroimaging 1 (5), 433–447. 10.1016/j.bpsc.2016.04.002 27642641 PMC5013873

[B45] McDougal R. A. Morse T. M. Carnevale T. Marenco L. Wang R. Migliore M. (2017). Twenty Years of ModelDB and beyond: Building Essential Modeling Tools for the Future of Neuroscience. J. Comput. Neurosci. 42 (1), 1–10. 10.1007/s10827-016-0623-7 27629590 PMC5279891

[B46] Medaglia J. D. Lynall M.-E. Bassett D. S. (2015). Cognitive Network Neuroscience. J. Cogn. Neurosci 27 (8), 1471–1491. 10.1162/jocn_a_00810 25803596 PMC4854276

[B47] Meisel C. (2020). Antiepileptic Drugs Induce Subcritical Dynamics in Human Cortical Networks. Proc. Natl. Acad. Sci. U.S.A. 117 (20), 11118–11125. 10.1073/pnas.1911461117 32358198 PMC7245074

[B48] Mujica-Parodi L. R. Amgalan A. Sultan S. F. Antal B. Sun X. Skiena S. (2020). Diet Modulates Brain Network Stability, a Biomarker for Brain Aging, in Young Adults. Proc. Natl. Acad. Sci. U.S.A. 117 (11), 6170–6177. 10.1073/pnas.1913042117 32127481 PMC7084077

[B49] Nagai Y. Aram J. Koepp M. Lemieux L. Mula M. Critchley H. (2018). Epileptic Seizures Are Reduced by Autonomic Biofeedback Therapy through Enhancement of Fronto-Limbic Connectivity: A Controlled Trial and Neuroimaging Study. Ebiomedicine 27, 112–122. 10.1016/j.ebiom.2017.12.012 29289531 PMC5828368

[B50] Nagai Y. (2019). Autonomic Biofeedback Therapy in Epilepsy. Epilepsy Res. 153, 76–78. 10.1016/j.eplepsyres.2019.02.005 30819542

[B51] Newman Mark. (2018). Networks. Oxford University Press.

[B52] Nicholas T. C. Michael L. H. (2010). The NEURON Book. Cambridge University Press.

[B53] Olson C. A. Vuong H. E. Yano J. M. Liang Q. Y. Nusbaum D. J. Hsiao E. Y. (2018). The Gut Microbiota Mediates the Anti-seizure Effects of the Ketogenic Diet. Cell 174 (2), 497. 10.1016/j.cell.2018.06.051 30007420 PMC6062008

[B54] Paz J. T. Huguenard J. R. (2015). Microcircuits and Their Interactions in Epilepsy: Is the Focus Out of Focus? Nat. Neurosci. 18 (3), 351–359. 10.1038/nn.3950 25710837 PMC4561622

[B55] Pitkänen A. Löscher W. Vezzani A. (2016). Advances in the Development of Biomarkers for Epilepsy. Lancet Neurol. 15 (8), 843–856. 10.1016/s1474-4422(16)00112-5 27302363

[B56] Pitkänen A. Lukasiuk K. Dudek F. E. Staley K. J. (2015). Epileptogenesis. Cold Spring Harb Perspect. Med. 5 (10), a022822. 10.1101/cshperspect.a022822 26385090 PMC4588129

[B57] Ramaraju S. Wang Y. Sinha N. McEvoy A. W. Miserocchi A. de Tisi J. (2020). Removal of Interictal MEG-Derived Network Hubs Is Associated with Postoperative Seizure Freedom. Front. Neurol. 11, 563847. 10.3389/fneur.2020.563847 33071948 PMC7543719

[B58] Rho J. M. (2017). How Does the Ketogenic Diet Induce Anti-seizure Effects? Neurosci. Lett. 637, 4–10. 10.1016/j.neulet.2015.07.034 26222258

[B59] Rings T. Von Wrede R. Bröhl T. Schach S. Helmstaedter C. Lehnertz K. (2021). Impact of Transcutaneous Auricular Vagus Nerve Stimulation on Large-Scale Functional Brain Networks: From Local to Global. Front. Physiol. 12, 700261. 10.3389/fphys.2021.700261 34489724 PMC8417898

[B60] Rings T. von Wrede R. Lehnertz K. (2019). Precursors of Seizures Due to Specific Spatial-Temporal Modifications of Evolving Large-Scale Epileptic Brain Networks. Sci. Rep. 9 (1), 10623. 10.1038/s41598-019-47092-w 31337840 PMC6650408

[B61] Rubinov M. Sporns O. (2010). Complex Network Measures of Brain Connectivity: Uses and Interpretations. Neuroimage 52 (3), 1059–1069. 10.1016/j.neuroimage.2009.10.003 19819337

[B62] Sanz Leon P. Knock S. A. Woodman M. M. Domide L. Mersmann J. McIntosh A. R. (2013). The Virtual Brain: a Simulator of Primate Brain Network Dynamics. Front. Neuroinform. 7 (MAY), 10. 10.3389/fninf.2013.00010 23781198 PMC3678125

[B63] Schach S. Rings T. Bregulla M. (2022). Electrodermal Activity Biofeedback Alters Evolving Functional Brain Networks in People with Epilepsy, but in a Non-specific Manner. Front. Neurosci. 16, 828283. 35310086 10.3389/fnins.2022.828283 PMC8927283

[B64] Schroeder G. M. Diehl B. Chowdhury F. A. Duncan J. S. de Tisi J. Trevelyan A. J. (2020). Seizure Pathways Change on Circadian and Slower Timescales in Individual Patients with Focal Epilepsy. Proc. Natl. Acad. Sci. U.S.A. 117 (20), 11048–11058. 10.1073/pnas.1922084117 32366665 PMC7245106

[B65] Sen A. Jette N. Husain M. Sander J. W. (2020). Epilepsy in Older People. The Lancet 395 (10225), 735–748. 10.1016/s0140-6736(19)33064-8 32113502

[B66] Sinha N. Dauwels J. Kaiser M. Cash S. S. Brandon Westover M. Wang Y. (2016). Predicting Neurosurgical Outcomes in Focal Epilepsy Patients Using Computational Modelling. Brain 140 (2), 319–332. 10.1093/brain/aww299 28011454 PMC5278304

[B67] Sinha N. Dauwels J. Kaiser M. Cash S. S. Brandon Westover M. Wang Y. (2017). Reply: Computer Models to Inform Epilepsy Surgery Strategies: Prediction of Postoperative Outcome. Brain 140 (5), e31. 10.1093/brain/awx068 28334902 PMC10448005

[B68] Sinha N. Dauwels J. Yujiang Wang Y. Cash S. S. Taylor P. N. (20143620). An In Silico Approach for Pre-surgical Evaluation of an Epileptic Cortex. Annu. Int. Conf. Ieee Eng. Med. Biol. Soc 2014, 4884–4887. 10.1109/embc.2014.6944718 25571086

[B69] Sinha N. Davis K. A. (2021). Mapping Epileptogenic Tissues in MRI-Negative Focal Epilepsy. Neurology 97 (16), 754–755. 10.1212/wnl.0000000000012696 34521690

[B70] Sinha N. Peternell N. Schroeder G. M. Tisi J. Vos S. B. Winston G. P. (2021). Focal to Bilateral Tonic-Clonic Seizures Are Associated with Widespread Network Abnormality in Temporal Lobe Epilepsy. Epilepsia 62 (3), 729–741. 10.1111/epi.16819 33476430 PMC8600951

[B71] Sinha N. Wang Y. Dauwels J. Kaiser M. Thesen T. Forsyth R. (2019). Computer Modelling of Connectivity Change Suggests Epileptogenesis Mechanisms in Idiopathic Generalised Epilepsy. NeuroImage: Clin. 21, 101655. 10.1016/j.nicl.2019.101655 30685702 PMC6356007

[B72] Sinha N. Wang Y. Moreira da Silva N. Miserocchi A. McEvoy A. W. de Tisi J. (2021). Structural Brain Network Abnormalities and the Probability of Seizure Recurrence after Epilepsy Surgery. Neurology 96 (5), 11315. 10.1212/wnl.0000000000011315 PMC7884990 33361262

[B73] Sloviter R. S. (2008). Hippocampal Epileptogenesis in Animal Models of Mesial Temporal Lobe Epilepsy with Hippocampal Sclerosis: The Importance of the “Latent Period” and Other Concepts. Epilepsia 49 (s9), 85–92. 10.1111/j.1528-1167.2008.01931.x 19087122

[B74] Spencer D. D. Gerrard J. L. Zaveri H. P. (2018). The Roles of Surgery and Technology in Understanding Focal Epilepsy and its Comorbidities. Lancet Neurol. 17 (4), 373–382. 10.1016/s1474-4422(18)30031-0 29553383

[B75] Spencer S. S. (2002). Neural Networks in Human Epilepsy: Evidence of and Implications for Treatment. Epilepsia 43 (3), 219–227. 10.1046/j.1528-1157.2002.26901.x 11906505

[B76] Stacey W. Kramer M. Gunnarsdottir K. Gonzalez-Martinez J. Zaghloul K. Inati S. (2020). Emerging Roles of Network Analysis for Epilepsy. Epilepsy Res. 159, 106255. 10.1016/j.eplepsyres.2019.106255 31855828 PMC6990460

[B77] Tang V. Michaelis R. Kwan P. (2014). Psychobehavioral Therapy for Epilepsy. Epilepsy Behav. 32, 147–155. 10.1016/j.yebeh.2013.12.004 24418662

[B78] Taylor P. N. Papasavvas C. A. Owen T. W. Schroeder G. M. Hutchings F. E. Chowdhury F. A. (2022). Normative Brain Mapping of Interictal Intracranial EEG to Localize Epileptogenic Tissue. Brain. Published online 2022:awab380-. 10.1093/brain/awab380 PMC9050535 35075485

[B79] Taylor P. N. Sinha N. Wang Y. Vos S. B. de Tisi J. Miserocchi A. (2018). The Impact of Epilepsy Surgery on the Structural Connectome and its Relation to Outcome. NeuroImage: Clin. 18, 202–214. 10.1016/j.nicl.2018.01.028 29876245 PMC5987798

[B80] Tecchio F. Cottone C. Porcaro C. Cancelli A. Di Lazzaro V. Assenza G. (2018). Brain Functional Connectivity Changes after Transcranial Direct Current Stimulation in Epileptic Patients. Front. Neural Circuits 12, 44. 10.3389/fncir.2018.00044 29899691 PMC5988884

[B81] Thijs R. D. Surges R. O'Brien T. J. Sander J. W. (2019). Epilepsy in Adults. The Lancet 393 (10172), 689–701. 10.1016/s0140-6736(18)32596-0 30686584

[B82] Thijs R. D. (2019). The Autonomic Signatures of Epilepsy: Diagnostic Clues and Novel Treatment Avenues. Clin. Auton. Res. 29 (2), 131–133. 10.1007/s10286-019-00603-1 30903303

[B83] Tsuboyama M. Kaye H. L. Rotenberg A. (2020). Review of Transcranial Magnetic Stimulation in Epilepsy. Clin. Ther. 42 (7), 1155–1168. 10.1016/j.clinthera.2020.05.016 32624320

[B84] van Loo K. M. J. Becker A. J. (2020). Transcriptional Regulation of Channelopathies in Genetic and Acquired Epilepsies. Front. Cel. Neurosci. 13, 587. 10.3389/fncel.2019.00587 PMC6971179 31992970

[B85] Vlachos I. Kugiumtzis D. Tsalikakis D. G. Kimiskidis V. K. (2022). TMS-induced Brain Connectivity Modulation in Genetic Generalized Epilepsy. Clin. Neurophysiol. 133, 83–93. 10.1016/j.clinph.2021.10.011 34814019

[B86] von Wrede R. Rings T. Schach S. Helmstaedter C. Lehnertz K. (2021). Transcutaneous Auricular Vagus Nerve Stimulation Induces Stabilizing Modifications in Large-Scale Functional Brain Networks: towards Understanding the Effects of taVNS in Subjects with Epilepsy. Sci. Rep. 11 (1), 7906. 10.1038/s41598-021-87032-1 33846432 PMC8042037

[B87] Wang Y. Sinha N. Schroeder G. M. Ramaraju S. McEvoy A. W. Miserocchi A. (2020). Interictal Intracranial Electroencephalography for Predicting Surgical success: The Importance of Space and Time. Epilepsia 61, 1417–1426. (January):epi. 10.1111/epi.16580 32589284 PMC7611164

[B88] Winston G. P. Cardoso M. J. Williams E. J. Burdett J. L. Bartlett P. A. Espak M. (2013). Automated Hippocampal Segmentation in Patients with Epilepsy: Available Free Online. Epilepsia 54 (12), 2166–2173. 10.1111/epi.12408 24151901 PMC3995014

[B89] Wolf P. Beniczky S. (2014). Understanding Ictogenesis in Generalized Epilepsies. Expert Rev. Neurotherapeutics 14 (7), 787–798. 10.1586/14737175.2014.925803 24875327

[B90] YueJ. K.YuhE. L.KorleyF. K.WinklerE. A.SunX.PufferR. C. (2019). Association between Plasma GFAP Concentrations and MRI Abnormalities in Patients with CT-negative Traumatic Brain Injury in the TRACK-TBI Cohort: a Prospective Multicentre Study. Lancet Neurol. 18 (10), 953–961. 10.1016/s1474-4422(19)30282-0 31451409

